# Influencing factors of residents’ environmental health literacy in Shaanxi province, China: a cross-sectional study

**DOI:** 10.1186/s12889-022-12561-x

**Published:** 2022-01-17

**Authors:** Yan Zhao, Yun Sheng, Jieting Zhou, Hao Wang, Mumba Mulutula Chilufya, Xuan Liu, Alaa Osman Mohamed, Jing Han, Chengjuan Qu

**Affiliations:** 1 School of Public Health, Health Science Center Xi’an Jiaotong University, Xi’an, Shaanxi 710061 PR China; 2 Shaanxi Provincial Academy of Environmental Science, Xi’an, Shaanxi 710061 PR China; 3 Department of Odontology, Umeå University, 90185 Umeå, Sweden

**Keywords:** Environmental health literacy, Influencing factor, Urban, Rural, Health promotion

## Abstract

**Background:**

This study comprehensively analyzed the basic conditions and influencing factors of the residents' environmental health literacy (EHL) level in Shaanxi Province, China in 2020, and provided a scientific basis for exploring new ideas and new methods to improve the EHL level of the whole people.

**Methods:**

In the cross-sectional study with a multi-stage random sampling method, 1320 participants were recruited in 6 neighborhood committees (administrative villages) from the Shaanxi province of China between 15–69 years old. The Core Questions for Assessment of EHL of Chinese Citizens (Trial Implementation) was adopted to measure the EHL of the respondents.

**Results:**

The survey showed the level of EHL of residents is 17.6% in Shaanxi in 2020. Among them, the basic concepts, basic knowledge, and basic skills classification literacy levels are 34.7%, 6.89%, and 37.95% respectively. The EHL ratio of rural residents is significantly lower than that of urban residents (12.38 *vs.* 29.02%). A noticeable difference was shown in various aspects and environmental health issues of EHL between urban and rural populations.

**Conclusions:**

Many factors are affecting the level of EHL. Education and science popularization of basic environmental and health knowledge in key areas and populations should be strengthened, and behavioral interventions should be carried out according to the characteristics of the population.

## Background

Environmental health literacy (EHL) is a wide range of skills and competencies that require people to seek out, comprehend, evaluate, and use environmental health information to make informed choices, reduce health risks, improve quality of life and protect the environment [1]. Environmental literacy (EL) involves more than just being informed about the environment, it also involves the ability to make environmentally-friendly decisions [2]. Health literacy (HL) refers to the ability of individuals to access health information and to make informed decisions about treatment and action for themselves and others [3, 4]. Increased HL will significantly improve personal and community health by changing personal lifestyles and living conditions [5]. The implementation of HL policies should be emphasized by health decision-makers and politicians so that health care providers can use their expertise and skills to coordinate health care more effectively around the needs of communities and populations [6]. However, the conceptualization of HL at the individual level is questionable, focusing only on secondary and tertiary prevention of disease, rather than primary prevention. Instead, the concept of public health literacy (PHL) is introduced, which highlights individuals and groups make public health decisions that benefit the community [7, 8].

EHL is a natural outgrowth of other literacies, including EL, HL, and PHL. As an emerging subdiscipline, EHL integrates key elements from various areas of study, including risk communication, environmental health sciences (EHS), and safety culture [9]. High EHL can potentially lead to a “greater understanding of specific risks, the reduction of exposures, and the improvement of health outcomes for individuals and communities” [10]. Meanwhile, existing studies suggest that many chronic health conditions are related to environmental contamination [11–13]. EHL is generally regarded as a dynamic process through which individuals and communities are increasingly able to have an understanding of environmental and health (EH) risks, exposures, outcomes, and strategies to reduce adverse environmental exposures and promote health [14].

At present, there is little research on the monitoring of EHL at home [15] and abroad [16], especially through theoretical and practical methods, making this study all the more important and timely. In China with a population of approximately 1.4 billion [17], based upon recent developments in this nascent field, namely: the 2013 Environmental Health Literacy of Chinese Citizens Trial Implementation (Announcement No. 61 2013) which introduced the contents of basic concepts, basic knowledge, and basic skills [15]; the 2016 results of the sample survey on EHL of residents released by Chinese Society of Environmental Sciences, only 8.41%, suggesting that efforts should be made to popularize and disseminate EH knowledge and improve the level of EHL among the population [18]; as well as the "Technical Guidelines for Evaluation of Citizens' (Trial)" (Announcement No. 24 of 2017) [19], this study was formulated and executed.

The goal of the study is to understand the level of EHL and its influencing factors at this stage, focus on the disparity between the EHL levels of urban areas in Shaanxi versus the rural areas. In addition, this study seeks to compare the national baseline level for EHL as it was at the first domestic large-scale monitoring effort [20] to the level in Shaanxi province. With this information and context, more targeted recommendations can be made for the improvements of the EHL of the populace, and with these improvements, citizens can find themselves both informed and empowered to engage in environmental protection and preservation efforts as well as protect themselves from some environmental dangers [21]. With the more concentrated and advised intervention of government bodies, combined with the better-educated actions of citizens, there can be amelioration and aversion, both of environmental damage and of environmental hazards, such as climate change, stratospheric ozone depletion, biodiversity loss, changes in hydrological systems, and the supply or depletion of freshwater, land degradation bring [22].

## Methods

### Study population and sampling design

This study was a cross-sectional survey conducted in Shaanxi, China in 2020. The study population (38.76 million) [23] was permanent residents aged 15 to 69 years in 6 prefecture-level cities. This population had lived in the area for at least 6 months in the past 12-month period, regardless of whether they had a local household registration. However, the residents who lived in hospitals, prisons, nursing homes, dormitories, and other such places were excluded from the study. The study population only included residents of the People’s Republic of China.

**Fig. 1:**
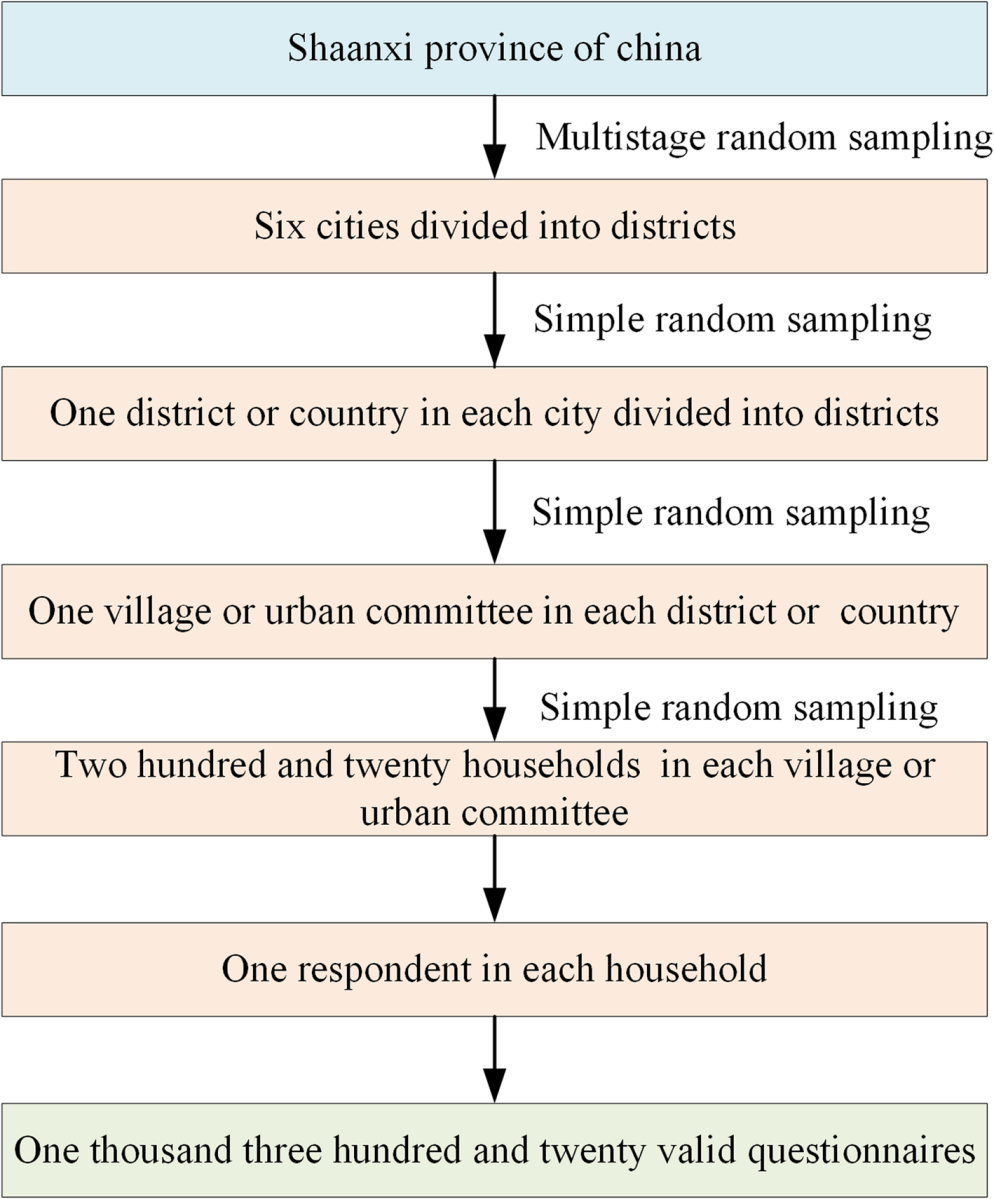
Flow chart for participants sampling in the study on environmental health literacy in a 15 to 69-year-old population, Shaanxi, China, 2020

A multi-stage cluster sampling method was adopted to select participants. The advantage of cluster sampling is that it can fully ensure the consistency of sample structure and population while enhancing the representativeness of samples. The process is shown in Fig. 1. The sample size of each selected urban resident committee or village committee was calculated by

$${\text{n}} = \left( {z_{a}^{2} \times p\left( {1 - p} \right)} \right)/e^{2} \times deff\left( 1 \right)$$

where α denotes the significance level; z_α_ is the value of z when α is equal to 0.05; *p* is the percentage of people with EHL; e is the maximum permissible error; deff represents the design effect of complex sampling adopted to adjust the loss of effectiveness due to complex sampling instead of simple random sampling. A 95% confidence limit was set, p was usually 0.5, the relative error rate was between 10 and 20%, the usual value of deff ranged from 1.5 to 2.0. Calculated according to the above formula, the minimum sample size of each layer ranges from 192.08 to 576.24, so the minimum sample size for this study is 220. The total sample size was calculated by

$$N = n\left( {n_{FPC} } \right) \times \left( {product\;of\;stratification\;factors} \right) \times \left( {1 + refusal\;rate} \right)$$

where n or n_FPC_ is the minimum sample size for each layer; the product of stratification factors denotes that the urban and rural areas have 2 levels, and the gender has 2 levels, and the product is 4 in this study; the refusal rate is 8%. Lastly, the total expected sample capacity of Shaanxi province was 192.08 × 4 × (1 + 8%) = 830 and 576.24 × 4 × (1 + 8%) = 2489 in 6 prefecture-level cities. According to actual conditions, 1375 people from 6 regions (3 urban areas and 3 rural areas) of Shaanxi Province were finally selected in the study. However, a total of 1320 people were included in the final analysis after data cleaning [24] due to some missing critical information (address, gender, and age) or EHL outcome variables.

### Data collection and questionnaire survey

**Fig. 2:**
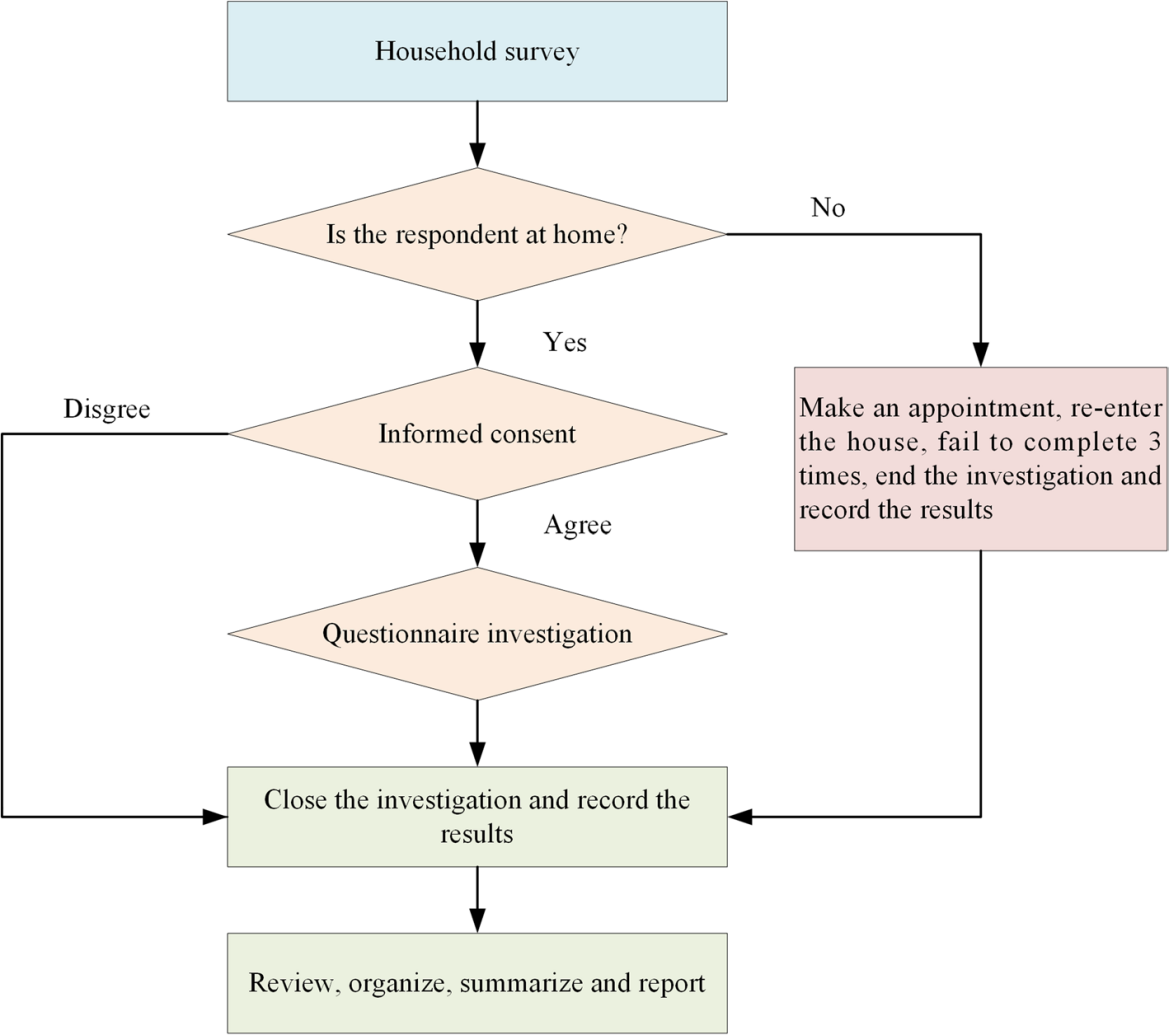
Flow chart for household investigation in the study on environmental health literacy in a 15 to 69-year-old population, Shaanxi, China, 2020

All participants completed written informed consent forms prior to the study. These questionnaires were collected in households by unified trained investigators. The flow chart of household investigation is given in Fig. 2. The questionnaire is divided into two parts: the first part was aimed at collecting socio-demographic characteristics (e.g., gender, age, education level, and occupation), and the second part evaluated the EHL level based on the “The Core Questions For Assessment of EHL of Chinese Citizens (Trial Implementation)” developed by the Chinese Ministry of Ecological Environment [19]. The Core Questions contains 47 questions, including 13 judgment questions, 15 single-response questions, and 19 multiple-response questions. Judgment questions: put a “√” or an “x” in the brackets for the corresponding question; Single-response questions: for each question, there are 4 options, only 1 of which is the correct answer, tick the corresponding option. If you do not know the answer, but a “√” directly on option ④; Multiple-response questions: there are 5 options for each question, of which 2 or more are correct, tick the corresponding option. If you do not know the answer, put a tick on option ⑤.

The content of the EHL evaluation is divided into three first-level categorical literacy (e.g., basic concepts, basic knowledge, and basic skills), every first-level indicator is further divided into two second-level indicators (e.g., basic cognition, basic attitude, scientific knowledge, behavioral knowledge, cognitive skills, as well as operating skills). The basic concepts emphasize correct knowledge and scientific understanding of the relationship between the environment and health, highlighting the concept of prevention and awareness of responsibility; basic knowledge covers air, water, soil, sea, biodiversity, climate change, radiation, and noise; basic behaviors and skills include green and healthy lifestyles and behaviors, as well as skills in acquiring, understanding and applying relevant information and in emergency response, monitoring and safeguarding rights. The questionnaire's half reliability of 0.729 and Cronbach's alpha coefficient of 0.965 (0.921, 0.844, and 0.814 for the basic concepts, knowledge, and skills sections respectively) indicate that the internal consistency of the questionnaire is good and that it is a reliable reflection of the level of EHL and can measure EHL of citizens [25].

### Score criteria

The total score of the questionnaire is 100 points, with 13 for judgment questions, 30 for single-response questions, and 57 for multiple-response questions. Basic concepts (question 1、2、14、15、16、17、29、30、31、35、36、37、38), basic knowledge (question 3、4、5、6、7、13、18、19、20、22、32、33、34、39、40、41、42、43), and basic skills (question 8、9、10、11、12、21、23、24、25、26、27、28、44、45、46、47) are 31、38、31 respectively. The scores of basic cognition (question 1、2、14、15、29、35、36、37), basic attitude (question 16、17、30、31、38), scientific knowledge (question 3、4、5、6、7、13、19、20、22、34、40、41), behavioral knowledge (question 18、32、33、39、42、43), cognitive skills (question 9、10、12、21、24、26、27、28、45), as well as operating skills (question 8、11、23、25、44、46、47) are 18、13、21、17、16、15. The scoring criteria are: Judgment problem, correct count 1, error count 0; Single-response, correct count 2, error count 0; Multiple-response, completely correct count 3, and wrong choice or missed choice count 0. The questionnaire score of 70% or more (≥ 70) is used as a criterion for determining whether a particular respondent has EHL, while the overall level of EHL refers to the percentage of people judged to be EHL with the total number of people surveyed. Similarly, categorical literacy is judged as the sum of the scores for all questions on a dimension and the actual score of 70% or more of the total score for that dimension. The level of categorical literacy refers to the proportion of the total number of people in the survey area who have a particular dimension of literacy.

### Statistical analysis

Statistical analysis was conducted using SPSS version 18.0 (IBM, Armonk, NY, USA). Basic socio-demographic variables were described by descriptive statistics. Chi-square bivariate tests were performed to determine the group differences (having basic EHL or not) for all demographic variables. The EHL-related data were weighted according to the seventh national census and due to this being a cross-sectional survey, the method of complex sampling [24] was adopted. Chi-square tests were used to compare the EHL level in various aspects and environmental health issues between urban and rural populations. Linear regression was aimed to describe the relationship between the level of EHL and the basic concepts, basic knowledge, and basic skills. Finally, binary logistic regression was conducted to verify if socio-demographic and environmental health variables are associated with EHL levels in Chinese residents. When *P*-value was less than 0.05 (two-tailed), the difference is considered to be statistically significant.

## Results

### Demographic characteristics and EHL status

The average age of the respondents was (39.86 ± 13.53) years. For all respondents, the ratio of males to females was 1:1, urban and rural residents were also equally divided (Table 1). Those aged 15–24 accounted for 13%, aged 25–34 accounted for 27%, aged 35–44 accounted for 22%, aged 45–54 accounted for 21%, aged 55–64 accounted for 13%, and aged 65–69 occupied 4%. In the region, most respondents had achieved at least a middle school-level education. In addition, the annual income of most respondents exceeded RMB 5500. After data weighted adjustment, the level of EHL in the residents was 17.60%. The level of EHL in urban populations was significantly higher than that in rural populations (29.02% vs. 12.38%). The EHL level in males was significantly higher than that in females (22.47% vs. 16.01%). The EHL level of people aged 25–34 was significantly higher than other age groups. The level of EHL was significantly higher in those better educated (Table 1).

**Table 1:** Sample characteristics and level of environmental health literacy in the participants (*n* = 1320)

Variable	Category	Number of Survey	Level of EHL			^2^	*P*
			***n***	**Sample Rate (%)**	**Weighted Rate (%)**		
Region	Urban	660	165	25.00	29.02	40.208	< 0.001
	Rural	660	76	11.51	12.38		
Gender	Male	660	135	20.45	22.47	4.269	0.039
	Female	660	106	16.06	16.01		< 0.05
Age groups	15–24	171	32	18.71	19.53	37.777	< 0.001
	25–34	357	91	25.49	25.66		
	35–44	288	61	21.18	24.09		
	45–54	284	30	10.56	10.95		
	55–64	173	24	13.87	13.72		
	65–69	47	3	6.38	6.32		
Education level	Primary school and below	133	4	3.01	1.71	190.294	< 0.001
	Junior high school	339	21	6.19	4.67		
	Senior high school/Technical secondary school/Vocational school	307	39	12.7	12.62		
	Junior college	257	61	23.74	24.27		
	Undergraduate	219	75	34.20	37.05		
	Postgraduate or above	65	41	63.08	64.07		
Annual income level	< 5500	272	32	11.80	11.27	36.689	< 0.001
	5500–12,999	399	60	15.03	16.06		
	13,000–20,999	184	36	19.57	17.62		
	21,000–31,999	122	16	13.11	13.58		
	> 32,000	332	95	28.61	32.80		
Total		1320	241	18.26	17.6		

### The differences of first-level classification literacy between urban and rural residents

**Fig. 3:**
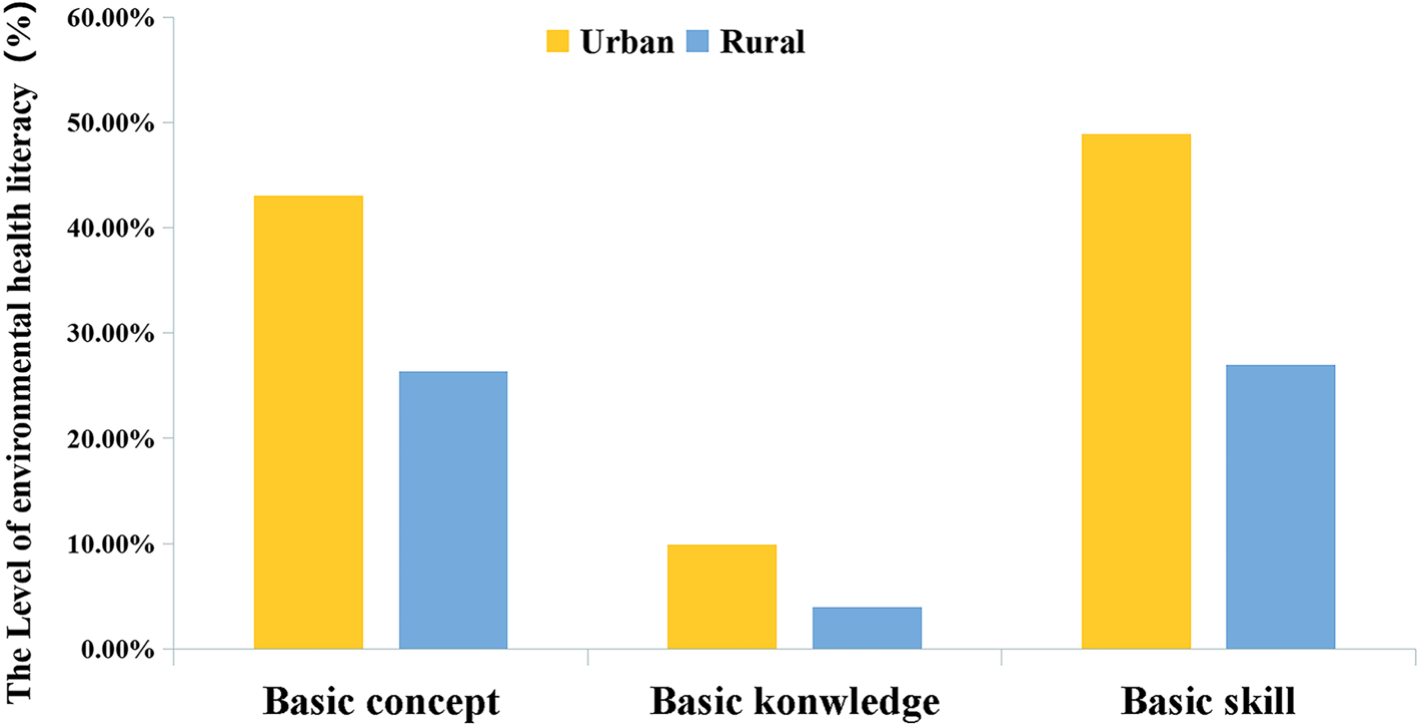
The level of EHL in 3 different aspects (i.e., basic knowledge, basic concepts, and basic skills) in urban and rural populations

Figure 3 presents the level of first-level categorical EHL of urban and rural residents. Whether in rural or urban areas, the level of EHL in basic knowledge was relatively low, and the level of EHL in basic skills was higher than the level of EHL in basic concepts. As revealed from the results of chi-square tests, the awareness rate of basic concepts (43.03% *vs.* 26.36%), the possession rate of basic knowledge (9.85% *vs.* 3.94%), and the mastery rate of basic skills (48.94% *vs.* 26.97%) of urban residents were significantly higher than those in rural residents (***P*** < 0.001).

### The differences of second-level classification literacy between urban and rural residents

**Fig. 4:**
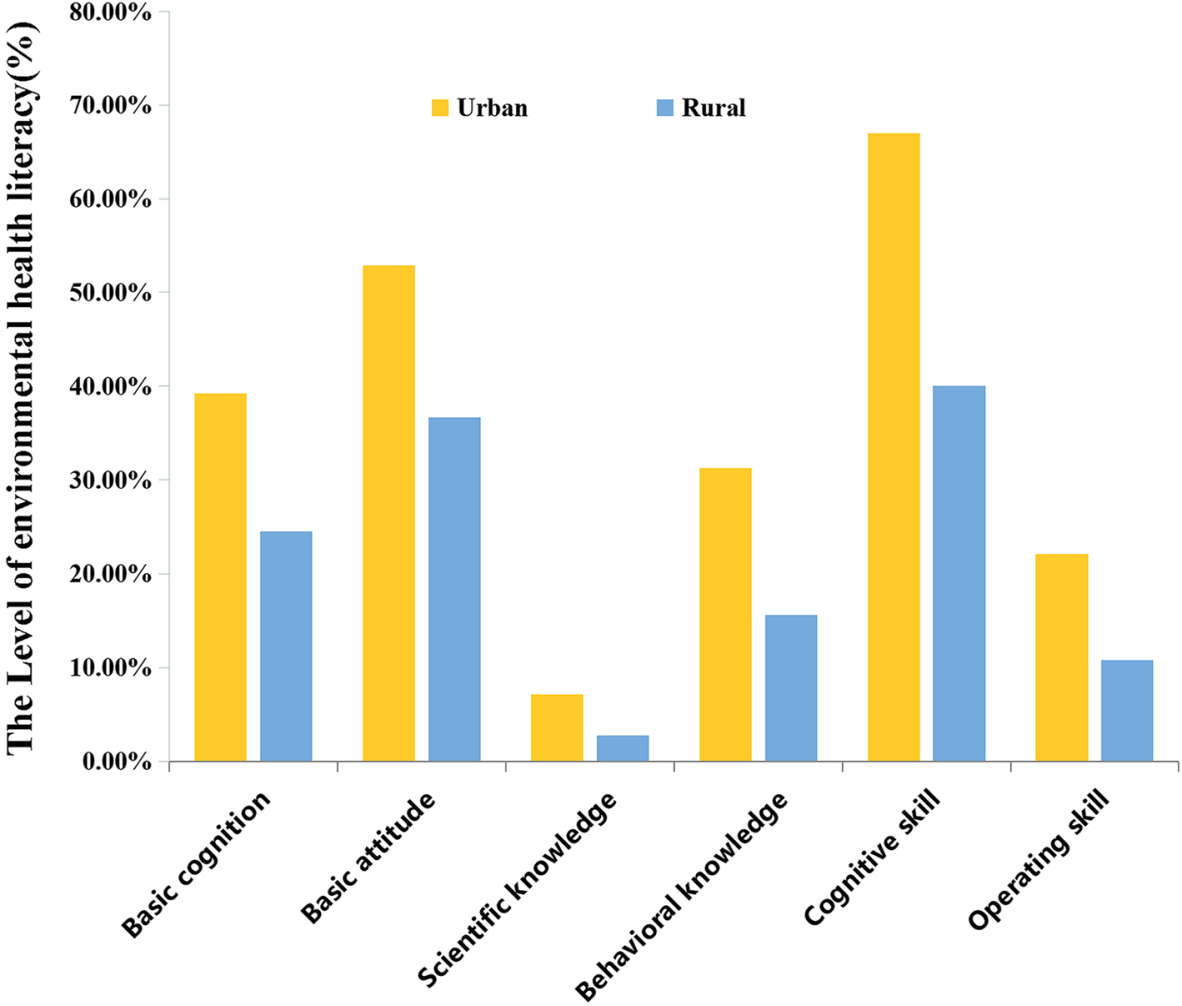
The level of environmental health literacy in 6 types of health problems: basic cognition, basic attitude, scientific knowledge, behavioral knowledge, cognitive skill, operating skill

Figure 4 shows the level of second-level categorical EHL of urban and rural residents. It can be observed that the EHL level of scientific knowledge was the lowest, and the EHL level of cognitive skills was higher than the level of EHL difference in other dimensions of environmental health issues in both rural and urban populations. Moreover, the EHL level of the different dimensions of environmental health issues, such as basic cognition (39.24% *vs.* 24.54%), basic attitude (52.88% *vs.* 36.67%), scientific knowledge (7.12% *vs.* 2.73%), behavioral knowledge (31.21% *vs.* 15.61%), cognitive skills (66.97% *vs.* 40.00%), and operating skills (22.12% *vs.* 10.76%) was significantly higher in urban residents than those in rural residents (*P* < 0.001).

### Linear regression analysis of the dimensions of EHL

Linear regression analysis was performed with a total score of EHL as the dependent variable and the basic concepts, basic knowledge, and basic skills as independent variables. The regression model is statistically significant, F = 7673.512 (*P* < 0.001), indicating that there is a relation between the dependent variable and the independent variable. According to the standard regression coefficients, it can be judged that the basic concepts in the three dimensions affect the EHL level more than basic knowledge and basic skills. The specific order of their impact is as follows: basic concepts (0.415) > Basic skills (0.352) > Basic knowledge (0.341) (Table 2).

**Table 2:** The linear regression relationship between the total score of EHL and its three dimensions

Dimensions	Unstandardized Coefficients	Standardized Coefficients	t	*P*
Constant	1.285		3.392	0.001
Basic knowledge	0.916	0.341	36.956	< 0.001
Basic skills	0.993	0.352	39.131	< 0.001
Basic concepts	1.012	0.415	43.582	< 0.001

Dependent variable: the total score of EHL

### The factors affecting EHL of urban and rural dwellers

In a binary logistic regression model, the first category of age groups, education level, and occupation is applied as a reference. After all other influencing factors were regulated in the logistic regression model, it was found that in urban areas, residents with an education from junior-senior high school / technical or secondary school / vocational school [OR = 8.245, 95% CI (1.770, 38.418)], associate degree[OR = 19.138, 95% CI (4.031, 90.864)], junior college [OR = 21.529, 95% CI (4.491, 103.215)], and postgraduate and above [OR = 40.338, 95% CI (7.532, 216.035)] achieved higher odds to exhibit basic EHL than those residents who only received a primary school level education and below. Residents aged from 35–44 [OR = 7.168, 95% CI (1.368, 37.571)] were more likely to exhibit basic EHL. Residents who were the production and auxiliary personnel in agriculture, forestry, animal husbandry, fishery, and water conservation industries [OR = 3.212, 95% CI (1.189, 8.678)], had higher odds to exhibit basic EHL. The results of entering logistic regression analyses are listed in Table 3.

**Table 3:** Odds ratios (OR) in favor of having basic EHL and 95% CI in the enter logistic regression

Risk factor	Urban			Rural	
	**OR**	**(95% CI)**	***P***	**OR (95% CI)**	***P***
Age groups					
15–24	Reference				
25–34	2.496 (0.462, 13.482)		0.288	1.358 (0.274, 6.746)	0.708
35–44	7.168 (1.368, 37.571)		0.020	1.311 (0.324, 5.315)	0.704
45–54	2.620 (0.493, 13.926)		0.259	1.530 (0.375, 6.238)	0.553
55–64	2.777 (0.518, 14.890)		0.233	0.000 (< 0.000)	0.996
65–69	5.315 (0.926, 30.502)		0.061	0.000 (< 0.000)	0.997
Education level					
Primary school and below	Reference				
Junior high school	2.486 (0.508, 12.155)		0.244	2.360 (0.486, 11.459)	0.287
Senior high school/Technical secondary school/Vocational school	8.245 (1.770, 38.418)		0.008	3.163 (0.545, 18.362)	0.200
Associate degree	19.138 (4.031, 90.864)		0.000	2.612 (0.371, 18.404)	0.335
Junior college	21.529 (4.491, 103.215)		0.000	4.689 (0.607, 36.254)	0.139
Postgraduate and above	40.338 (7.532, 216.035)		0.000	12.158 (0.596, 247.962)	0.104
Occupation					
Clerical and related personnel	Reference				
Party and state organs, mass organizations, social organizations, enterprises and public institutions	0.676 (0.336, 1.359)		0.271	1.317 (< 0.000)	0.997
Soldier	1.412 (0.656, 3.037)		0.378	0.969 (< 0.000)	1.000
The emeritus and retired	0.000 (< 0.000)		1.000	1.545 (< 0.000)	1.000
Production and auxiliary personnel in agriculture, forestry, animal husbandry, fishery and water conservation industries	3.212 (1.189, 8.678)		0.021	1.560 (< 0.000)	1.000
Social production service and life service personnel	0.489 (0.126, 1.895)		0.301	1.959 (< 0.000)	0.997
Manufacturing and related personnel	0.597 (0.240, 1.484)		0.267	6.572 (< 0.000)	0.997
Student	1.190 (0.454, 3.116)		0.724	1.402 (< 0.000)	0.997
Professionals	1.232 (0.568, 1.035)		0.597	1.504 (< 0.000)	1.000
Others	1.897 (1.035, 3.476)		0.038	7.304 (< 0.000)	0.997

## Discussion

This study assesses the level of EHL and its influencing factors among residents of Shaanxi province, China in 2020. According to the results of the study, the overall EHL level is low, and there are significant differences between different groups, and the overall improvement of the EHL level of residents needs to focus on youth, elderly and rural residents, and the low level of scientific knowledge literacy is a key issue that needs attention in the work of improving the EHL level of residents.

Results released that the EHL level is higher in Shaanxi China (17.6%) in 2020 than the 15% and above in 2022 required in the Healthy China Action (2019–2030) [20], but there is still a long way to go before reaching 25% and above in 2030. The level of rural residents is significantly lower than that of urban residents (12.38% *vs.* 29.02%), keeping with the results of the first survey of residents’ EHL in China (8.1% *vs.* 16.9%) [26]. The binary logistic regression results demonstrate that EHL is closely related to age, education level, occupation, and it is relatively low among residents of rural, females, low-educated, low income, and high-age groups. For instance, the EHL level of residents in Qinghai Province, which is relatively underdeveloped, was significantly lower than in the more developed Hubei Province (7.01% *vs.* 18.2%) in 2018 [27]. When carrying out relevant work, the local government can start with education and economics. Carry out educational reform and economic development in parallel, promote and communicate environmental and health (EH) issues to improve EHL and sustainable development.

According to the results of the linear regression analysis, the magnitude of the influence of the 3 dimensions on the level of EHL was ranked as basic knowledge > basic concepts > basic skills, with basic knowledge having the greatest influence. This should be given high priority by the organization to strengthen EH education, especially for basic EH knowledge. The overall mastery rate of basic knowledge is the lowest among the three dimensions of EHL, only 3.94%, which is far lower than 34.7% for basic concepts and 37.95% for basic skills. However, knowledge is in the first position in the chain of "knowledge-trust-action", according to the Knowledge, Attitude / Belief, Practice (KAP) Model [28]. Inadequate basic knowledge inevitably affects the formation of basic concepts and the acquisition of basic skills; the formation and development of behavior need to be based on the dissemination of basic knowledge and the formation of basic beliefs, and actual behavior can only be consolidated once knowledge of the possible benefits of behavior or the losses of not acting is known, and behavior change is supported by the acquisition of skills. It is, therefore, necessary that government and communities should vigorously promote the dissemination of EH knowledge. Strengthen residents’ environmental health education, especially in rural areas, so it can help people acquire more EHL-related knowledge, and thus change environmental and health-related behavior. Collective improvements in EHL will hopefully lead to positive health effects for individuals as well as for their families and the environment at large.

The questions on basic concepts, knowledge, skills and the specific indicators behind them are most closely related to the public’s daily life, current prominent EH issues and public opinion, covering the basic scientific concepts of environment and health, relevant scientific knowledge that highlights the health hazards related to environmental pollution issues (including scientific knowledge about the pollution causes, sources, exposure pathways, susceptible populations, major hazards, and behavioral measures for prevention or intervention, etc.), obtain and understand relevant information, and skills for complaints and rights protection, etc. These contents are either of interest to the public or have a strong link to the public's understanding of EH issues, protecting themselves from the health hazards of environmental pollution, and supporting the environment and health work. Assessing these components will provide a better basis for interventions and help to integrate EHL assessment with EH management.

### Limitations

This study has some limitations. Firstly, The Chinese Society of Environmental Sciences tested the reliability and validity of the questionnaire in a large sample of people through a preliminary survey, but it has not yet been verified in this specific area of Shaanxi province and needs to be carried out in the future. Secondly, the data in this study came from a cross-sectional survey, and this restricts the interpretation of the results of this study, making it difficult to draw general conclusions.

## Conclusion

In conclusion, through the survey and analysis of the EHL levels of residents in Shaani Xi province in 2020, it is possible to understand that the EHL levels of different groups of people. The data and analysis derived from the paper can provide some scientific guidance directions for future EHL popularization work. Based on the socio-demographic characteristics of each population group, targeted work programs will be developed to help the residents of Shaani Xi province to improve their EHL levels and enhance their quality of life.

## Data Availability

The datasets used and/or analyzed during the current study are available from the corresponding author on reasonable request.

## Abbreviations

EHL: Environmental health literacy
EL: Environmental literacy
HL: Health literacy
PHL: Public health literacy
EHS: Environmental health sciences
EH: Environmental and health

## References

[1] Marsili D, Comba P, De Castro P (2015). Environmental health literacy within the Italian Asbestos Project: experience in Italy and Latin American contexts. Commentary Ann Ist Super Sanita.

[2] O’Neil JM, Newton RJ, Bone EK, Birney LB, Green AE, Merrick B, Goodwin-Segal T, Moore G, Fraioli A (2020). Using urban harbors for experiential, environmental literacy: Case studies of New York and Chesapeake Bay. Reg Stud in Mar Sci.

[3] Katz A (2017). Health Literacy: What Do You Know?. Oncol Nurs Forum.

[4] Sorensen K, Van den Broucke S, Fullam J, Doyle G, Pelikan J, Slonska Z, Brand H (2012). Consortium Health Literacy Project E: Health literacy and public health: a systematic review and integration of definitions and models. BMC Public Health.

[5] Sany SBT, Doosti H, Mahdizadeh M, Orooji A, Peyman N (2021). The Health Literacy Status and Its Role in Interventions in Iran: A Systematic and Meta-Analysis. Int J Env Res Pub He.

[6] Sany SBT, Behzhad F, Ferns G, Peyman N (2020). Communication skills training for physicians improves health literacy and medical outcomes among patients with hypertension: a randomized controlled trial. Bmc Health Serv Res.

[7] Gray KM (2018). From Content Knowledge to Community Change: A Review of Representations of Environmental Health Literacy. Int J Env Res Pub He.

[8] Freedman DA, Bess KD, Tucker HA, Boyd DL, Tuchman AM, Wallston KA (2009). Public Health Literacy Defined. Am J Prev Med.

[9] Miller MD, Valenti M, Schettler T, Tencza B (2016). A Multimedia E-Book-A Story of Health: Filling a Gap in Environmental Health Literacy for Health Professionals. Environ Health Perspect.

[10] Raufman J, Blansky D, Lounsbury DW, Mwangi EW, Lan Q, Olloquequi J, Hosgood HD (2020). Environmental health literacy and household air pollution-associated symptoms in Kenya: a cross-sectional study. Environ Health.

[11] Chowdhury R, Ramond A, O'Keeffe LM, Shahzad S, Kunutsor SK, Muka T, Gregson J, Willeit P, Warnakula S, Khan H (2018). Environmental toxic metal contaminants and risk of cardiovascular disease: systematic review and meta-analysis. BMJ.

[12] Park SS, Skaar DA, Jirtle RL, Hoyo C (2017). Epigenetics, obesity and early-life cadmium or lead exposure. Epigenomics.

[13] Ruiz D, Becerra M, Jagai JS, Ard K, Sargis RM (2018). Disparities in Environmental Exposures to Endocrine-Disrupting Chemicals and Diabetes Risk in Vulnerable Populations. Diabetes Care.

[14] Lichtveld MY, Covert HH, Sherman M, Shankar A, Wickliffe JK, Alcala CS (2019). Advancing Environmental Health Literacy: Validated Scales of General Environmental Health and Environmental Media-Specific Knowledge, Attitudes and Behaviors. Int J Environ Res Public Health.

[15] Ministry of Ecological Environment of the People’s Republic of China. Announcement on the Release of Environmental Health Literacy of Chinese Citizens (Trail Implementation) [EB/OL] [http://www.mee.gov.cn/gkml/hbb/bgg/201310/t20131009_261336.htm]

[16] Finn S, O'Fallon L (2017). The Emergence of Environmental Health Literacy-From Its Roots to Its Future Potential. Environ Health Perspect.

[17] Communique of the National Bureau of Statistics of People’s Republic of China on Major Figures of the 2010 Population Census (No. 1) [http://www.stats.gov.cn/tjsj/pcsj/ rkpc/6rp/indexch.htm]

[18] Results of the First Survey of Chinese Residents' Environmental Health Literacy[EB/OL][http://www.mee.gov.cn/ywgz/fgbz/hjyjk/202008/t20200810_793281.shtml]

[19] Ministry of Ecological Environment of the People’s Republic of China. Announcement on the Release of Environment and Health Literacy of Chinese Citizens (Trial Implementation)[EB/OL] [http://www.mee.gov.cn/gkml/hbb/bgg/201310/t20131009_261336.htm]

[20] Action of Healthy China(2017–2030) [EB / OL] [http://www.gov.cn/xinwen/2019- 07/15/content_5409694.htm.]

[21] Landrigan PJ, Fuller R, Acosta NJR, Adeyi O, Arnold R, Basu NN, Balde AB, Bertollini R, Bose-O'Reilly S, Boufford JI (2018). The Lancet Commission on pollution and health. Lancet.

[22] Gray KM (2018). From Content Knowledge to Community Change: A Review of Representations of Environmental Health Literacy. Int J Environ Res Public Health.

[23] 2020 Statistical Yearbook [http://tjj.shaanxi.gov.cn/upload/n2020/indexch.htm]

[24] Li YHN, Xq NIE (2014). Contrast analysis of 2008 and 2012 Chinese health literacy survey scheme. Chin J Health Educ.

[25] Technical Guidelines for the Assessment of Citizen Environmental and Health Literacy (Trial) (Draft for Comments) [http://www.mee.gov.cn/gkml/hbb/bgg/201706/t20170608_415684.htm]

[26] Results of the First Survey of Chinese Residents' Environmental Health Literacy[EB/OL][ http://www.mee.gov.cn/ywgz/fgbz/hjyjk/gzdt/202008/t20200810_793281.shtml]

[27] Wang Qian LQ, Ailing H, Yi Y, Chunhua H, Liang S. Analysis on the current situation and influencing factors of environmental and health literacy of residents in Hubei Province. Environmental Science & Technology. 2020;43:230–6.

[28] editorial WCG (2012). It’s time to e-volve: taking responsibility for science communication in a digital age. Biol Bull.

